# Infection control at an urban hospital in Manila, Philippines: a systems engineering assessment of barriers and facilitators

**DOI:** 10.1186/s13756-017-0248-2

**Published:** 2017-09-02

**Authors:** Kaitlin F. Mitchell, Anna K. Barker, Cybele L. Abad, Nasia Safdar

**Affiliations:** 1 Department of Population Health Sciences, University of Wisconsin-Madison, Madison, WI USA; 2 Division of Infectious Diseases, Department of Medicine, University of Wisconsin-Madison, Madison, WI USA; 3 Department of Medicine, Division of Infectious Diseases, The Medical City, Pasig, Philippines; 4 William S. Middleton Memorial Veterans Hospital, Madison, WI USA; 5 Infection Control Department, University of Wisconsin-Madison, 5221 Medical Foundation Centennial Building, 1685 Highland Ave, Madison, WI 53705 USA

**Keywords:** Systems Engineering Initiative for Patient Safety, Philippines, Infection control, Hand hygiene, Intervention implementation

## Abstract

**Background:**

Healthcare facilities in low- and middle-income countries, including the Philippines, face substantial challenges in achieving effective infection control. Early stages of interventions should include efforts to understand perceptions held by healthcare workers who participate in infection control programs.

**Methods:**

We performed a qualitative study to examine facilitators and barriers to infection control at an 800-bed, private, tertiary hospital in Manila, Philippines. Semi-structured interviews were conducted with 22 nurses, physicians, and clinical pharmacists using a guide based on the Systems Engineering Initiative for Patient Safety (SEIPS). Major facilitators and barriers to infection control were reported for each SEIPS factor: person, organization, tasks, physical environment, and technology and tools.

**Results:**

Primary facilitators included a robust, long-standing infection control committee, a dedicated infection control nursing staff, and innovative electronic hand hygiene surveillance technology. Barriers included suboptimal dissemination of hand hygiene compliance data, high nursing turnover, clinical time constraints, and resource limitations that restricted equipment purchasing.

**Conclusions:**

The identified facilitators and barriers may be used to prioritize possible opportunities for infection control interventions. A systems engineering approach is useful for conducting a comprehensive work system analysis, and maximizing resources to overcome known barriers to infection control in heavily resource-constrained settings.

## Background

No health care facility in the world is immune to the burden of hospital-acquired infections (HAIs). Those in low- and middle-income countries such as the Philippines experience especially high rates of HAIs [1, 2], perhaps due to the added challenges they face in achieving effective infection control. These challenges include a higher prevalence of multi-drug resistant organisms (MDROs), lack of HAI surveillance, antibiotic overuse and misuse, and international migration of their healthcare workforce [3–5]. Assessing and improving the quality of infection control policies, hand hygiene, and HAI surveillance in these settings is critical [6].

In order to develop effective interventions, it is essential to understand how the work system in a healthcare setting may impede successful implementation [7]. The Systems Engineering Initiative for Patient Safety, or SEIPS framework, is well suited for its ability to analyze the impacts of a work system on both patient and organizational outcomes [8]. The work system includes the components of person (e.g. skills, motivation, and needs), tasks (e.g. job content), tools and technologies (e.g. information technologies or medical devices), the physical environment (e.g. layout and work station design), and organizational components (e.g. patient safety culture and communication). This model has been used to improve patient safety in a variety of healthcare contexts, including both outpatient and inpatient settings [9, 10].

Although the Philippines has a high HAI burden, an understanding of the facilitators and barriers to hospital infection control in this country is lacking [1–3]. To address this gap, we used the SEIPS framework to evaluate barriers and facilitators to infection control at a private, tertiary hospital located in Manila.

## Methods

### Location

The study was conducted at a private, 800-bed, tertiary hospital in Manila, Philippines. The facility is one of five hospitals in the Philippines accredited by the Joint Commission International. It employs 1000 physicians and over 2000 allied medical and administrative staff, and handles both routine and complex cases in many departments. The hospital has an infection control program that was established in 1986, and at the time of our study included six dedicated infection control nursing staff. Infection control policies are implemented and reviewed by the Hospital Infection Control Executive Committee. The facility has designated medical floors for those needing airborne isolation (e.g. pulmonary tuberculosis).

### Study population

We completed a total of 22 semi-structured interviews with physicians (*n* = 10; three male, seven female), nurses including infection control staff (*n* = 10; two male, eight female), and clinical pharmacists (*n* = 2; two female). Potential participants were selected by convenience sampling to cover a range of job types, experience levels, and clinical departments. Subjects who were available during the time investigators were conducting interviews were asked if they could participate and were identified on clinical wards by members of the research team. A few participants, including the two clinical pharmacists, were approached and recruited directly. Departments included cardiology, clinical pharmacy, gastroenterology, infection control, internal medicine, obstetrics and gynecology, oncology, pediatrics, and pulmonary medicine. Most participants worked in general wards in these departments, although two worked in the emergency department and two worked in the intensive care units (ICUs). Criteria for inclusion were formal employment by the hospital and active involvement in patient care. Status as a medical or nursing student or as a non-English speaker were criteria for exclusion, although no potential participants were excluded for these reasons.

### Data collection

Interview questions were adapted for context from an interview guide our group previously used to study facilitators and barriers to infection control at a large, private hospital in Gurgaon, India [11]. The questions were based on the SEIPS framework and included questions in the categories for work systems: person, organization, task, physical environment, and technology and tools. Interviews were audio recorded and typically lasted ten to twenty minutes in length. No identifiable information was collected. Preliminary analysis was conducted throughout the study to refine the interview guide and assess theoretical saturation. No further interviews were completed once theoretical saturation was reached.

### Data analysis

All interviews were transcribed. Interview transcripts were independently coded in NVivo software (Version 11, QSR International) by two individuals to identify trends, following a previously described method for line-by-line coding [12]. The two versions of coding were compared and found to have high inter-rater reliability. As a quality improvement project, this study was granted exemption from review by the UW-Madison Institutional Review Board and received expedited review and approval at the study site.

## Results

**Fig. 1:**
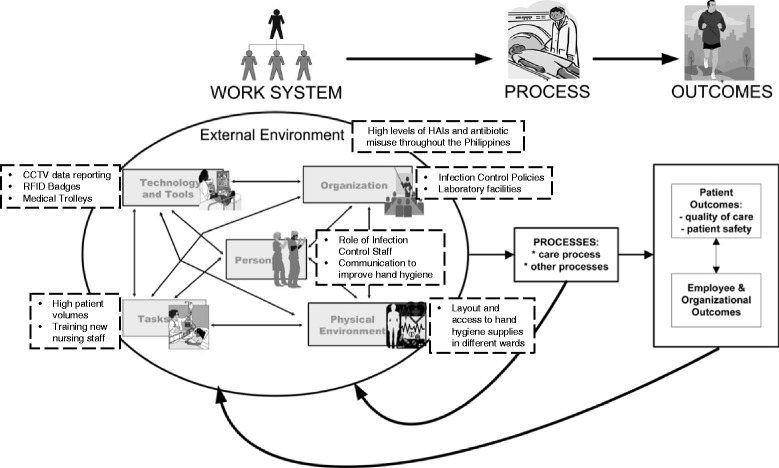
The SEIPS model, adapted from Carayon et al., 2006. [8] Themes identified during the present study are listed in boxes with dashed outlines. Themes are placed next to the corresponding component of the work system, including one identified theme related to the external environment (HAI, hospital-acquired infection; CCTV, closed circuit television; RFID, radio frequency identification)

Data were categorized based on the SEIPS model work system (Fig. 1 and Table 1).

**Table 1:** Representative quotations of themes identified using the SEIPS framework

SEIPS factor	Theme	Subject position and department	Quotation
Person	Role of Infection Control Staff	1: Staff Nurse, Infection Control	1: “We’re doing surveillance for infection control… like gathering data for patients who have risk devices. We’re also checking the environment… to make sure that infection control policies are implemented properly.”
		2: Staff Nurse, Oncology	2: “The infection control people visit us… they monitor us… they check everything on a daily basis.”
		3: Head Nurse, Internal Medicine	3: “[Healthcare workers are] amenable with the infection control rules… because they’re for the patients.”
	Communication to improve hand hygiene	4: Resident, Emergency Medicine	4: “It’s the nurses who do the hand hygiene more than the doctors”
		5: Resident, Pediatrics	5: “When [the consultants] go in and we follow, we forget to wash too.”
		6: Resident, Pediatrics	6: “I think it needs to be a team effort, like a constant reminder… the head nurses talk to you: ‘Doctor, you need to wash your hands’. I think it’s very helpful, you need to do that… But there are some doctors here who don’t really become too friendly with the nurses. They really set the barrier between them.”
Organization	Infection Control Policies	7: Staff Nurse, Infection Control	7: “HICEC is divided into committees… the executive committee makes the policies… we also have the nurses and staff from all different departments. Whenever we make a policy we make sure that all of [the representatives] approve.”
		8: Resident, first year	8: “It’s part of the infection control committee responsibilities to do regular lectures on hand hygiene.”
		9: Head Nurse, Internal Medicine	9: “We can coordinate with HICEC if we have a misunderstanding with the doctors… because we have a set of admitting guidelines for what is allowed in our ward, so sometimes we just have to tell the doctors that. You can also ask HICEC [to do that].”
	Laboratory Facilities	10: Attending Physician, Gastroenterology	10: “[The lab] immediately informs us… so that we can treat right away.”
		11: Resident, Pediatrics	11: “We try our best to prevent [resistant infections] here… but other doctors in other places, and the lay people, don’t have any idea what antibiotic resistance means.”
Tasks	High Patient Volumes	12: Resident, Emergency Medicine	12: “There are times we have to see one patient, just remove the gloves, and move onto the next patient, so there’s no [time for] alcohol in between.”
	Training of New Nursing Staff	13: Resident, first year	13: “[Nurses] get their training and then leave after about two to three years.”
		14: Head Nurse, Emergency Medicine	14: “We teach them all the standard precautions and diseases that any nurse could encounter.”
Physical Environment	Layout and Access to Hand Hygiene Supplies	15: Head Nurse, Internal Medicine	15: “The baby-friendly [obstetrics/gynecology ward] is much better with the hand hygiene because they have their own station there.”
		16: Resident, Emergency Medicine	16: “The alcohol rub is more in the station, where the medications are prepared [by the nurses]… so it’s not really that accessible to us.”
		17: Resident, Pediatrics	17: “I see some patients get the alcohol and place it in their room… one of the patients actually got the whole alcohol container.”
		18: Resident, Emergency Medicine	18: “There are some doctors who bring their own, or at least have it hooked on to them. And then that’s the time when they can do hand rubbing.”
Technology and Tools	CCTV Data Reporting	19: Head Nurse, Emergency Medicine	19: “[Each person] is not being reported, the whole emergency department is being reported. It’s not working I think, because when we look at the results they’re not specific.”
		20: Resident, Pediatrics	20: “They should call out those who don’t really hand wash, and talk to them directly. Because [the providers] don’t know they’re being monitored, so they think they can get away with it.”
	RFID Badges	21: Staff Nurse, Infection Control	21: “Every time the healthcare worker will enter the patient’s room, the ID will alarm if you don’t do hand hygiene.”
	Medical Trolleys	22: Head Nurse, Oncology	22: “The problem here is sometimes the nurses have too many things in their arms… [they need somewhere to] place things first while they’re rubbing their hands.”

*HICEC* hospital infection control executive committee, *CCTV* closed circuit television, *RFID* radio frequency identification

### Person

The multiple roles of the hospital’s infection control nursing staff were regarded as having a positive impact on the infection control process. They conducted HAI surveillance through review of medical records and bedside follow-up of high-risk patients. Infection control staff monitored hand hygiene compliance by performing daily audits through direct observation on each ward, and they also reviewed video footage of provider entry and exit from patient rooms using the hospital’s closed-circuit television system (Table 1, quotations 1 and 2). The infection control nurses and their duties were viewed with respect, and were regarded by other clinical staff as a vital component of the healthcare process (Table 1, quotation 3).

Another person-level factor was differing hand hygiene compliance between healthcare worker types. Both doctors and nurses reported that nurses had the highest hand hygiene compliance, while attending physicians had the lowest (Table 1, quotation 3). It was also noted that amongst the doctors, older consulting physicians tended to be the least compliant. This behavior was suggested to have a magnified impact on hand hygiene practices, as the senior physicians set “an example” for others (Table 1, quotation 5). Several doctors reported that verbal reminders, either from nurses or other physicians, would be a useful strategy to improve hand hygiene. However, it was also noted that the success of reminders would depend on the social dynamic between individual healthcare workers, as some attending physicians were more likely to be amenable to receiving feedback than others (Table 1, quotation 6).

### Organization

The hospital’s infection control executive committee was recognized as the organizational body that develops fundamental infection control guidelines such as those for hand hygiene, antimicrobial stewardship, and contact and other precautions. The representation of multiple departments on the committee was described as a necessary aspect of developing these guidelines (Table 1, quotation 7). These policies were monitored by the infection control staff, described above. The committee also organized promotional events and offered training for hand hygiene (Table 1, quotation 8). The committee and infection control guidelines could be called upon to resolve any disputes regarding appropriate patient care pertinent to infection control (Table 1, quotation 9). For example, they could be consulted for decisions regarding the appropriate placement of patients on the hospital’s isolation ward.

The hospital has prioritized funding and staffing for laboratory facilities, which were vital for performing tests to inform patient treatment plans and infection control surveillance. The turnaround time and communication of lab results through electronic reports were described favorably (Table 1, quotation 10). Efficient laboratory testing was thought to be particularly important for detecting antibiotic-resistant infections in patients who may have been exposed in the community, or another healthcare setting, prior to admission (Table 1, quotation 11).

### Task

There was considerable variation in the patient-to-provider ratios described across hospital departments, based on patient complexity and length of stay. The number of patients per nurse was estimated to be 1:1 or 2:1 in the ICUs, 5:1 or 6:1 in the obstetrics/gynecology and cancer wards, and approximately 16:1 in the cardiology ward. Several providers noted that a lack of time due to high patient volumes was a major barrier to hand hygiene compliance (Table 1, quotation 12).

Very high rates of nursing staff turnover also contributed to a high clinical workload. One nurse estimated that within the past six months, thirty out of ninety nurses had left the emergency department staff. The emigration of skilled healthcare workers from the Philippines was described as common; many nurses were motivated to move for higher-paying jobs abroad, leaving vacant positions that required continual resources to fill (Table 1, quotation 13). Most newly-hired nurses began jobs at this hospital directly out of nursing school, and needed a considerable amount of on-the-job infection control training (Table 1, quotation 14).

### Physical environment

Variations in the layout and quantity of hand hygiene supplies were believed to affect hand hygiene feasibility in certain departments. Alcohol-based hand rub dispensers were reported to be located outside of patient rooms, though the emergency department and ICU had additional dispensers located within patient rooms or cubicles. Sinks for handwashing were positioned at the nursing stations on most wards. However, the airborne isolation floor, emergency department isolation room, obstetrics/gynecology ward, and one select floor of private rooms had additional sinks within patient rooms. Several nurses described that the additional hand hygiene locations were an asset for those units (Table 1, quotation 15).

Multiple doctors stated that even though there were alcohol-based hand rub dispensers on every ward, these dispensers could not always be used conveniently (Table 1, quotation 16). Another barrier to the availability of hand hygiene supplies was the occasional theft of sanitizer or whole dispensers by patients, which was reported in multiple areas including the emergency department (Table 1, quotation 17). Several providers believed that the hospital could increase hand hygiene compliance by providing personal alcohol-based hand rub dispensers to each healthcare worker (Table 1, quotation 18).

### Technology and tools

This facility employed multiple technologies for hand hygiene auditing. A closed circuit television surveillance system was used to collect video footage of healthcare worker hand hygiene practices at the time of entry and exit from patient rooms. Although infection control nurses frequently reviewed the video footage, the data were not effectively communicated to clinical staff. Several doctors and nurses believed this footage was rarely or never reviewed. Others had received department-level feedback of hand hygiene compliance, but felt that reporting of compliance data to individual providers would be more helpful (Table 1, quotations 19 and 20).

Radio-frequency identification (RFID) badges utilized in the ICU were another innovative technology at this facility. These badges were detected by sensors on alcohol-based hand rub dispensers that recorded the duration and frequency of hand hygiene occurrences. The badges also provided instant reminder alarms for healthcare workers to perform hand hygiene (Table 1, quotation 21). All ICU nurses wore their own badge, allowing for individual compliance data to be tracked and reported in real-time on a television monitor prominently displayed in the ICU. However, visiting healthcare providers, including those that provided consults in the ICU, shared group RFID badges and their hand hygiene could not be monitored individually.

Medical trolleys were available on the airborne isolation ward of the hospital, and proved a useful tool for improving hand hygiene compliance for this area. The trolleys provided a place to set down medical supplies, making it easier for nurses to perform hand hygiene prior to entering the patient’s room. Several nurses expressed that having medical trolleys available on all wards would help improve hand hygiene throughout the hospital (Table 1, quotation 22). However, this would require the purchasing of trolleys out of the budget for each additional unit.

## Discussion

Our study design centered on the perspectives of healthcare providers to optimize future infection control interventions. Using the SEIPS model, we have framed multiple barriers and facilitators that were reported by nurses, doctors, and pharmacists at a private hospital in Manila, Philippines.

The long-established prioritization of an infection control program at this hospital is an organizational strength that is often lacking from the infrastructure of healthcare facilities in low- and middle-income countries [1, 13]. The representation of multiple departments on the program’s executive committee is aligned with a World Health Organization recommendation that hospital infection control policies be developed by a multidisciplinary team [14]. This program is especially important given the high prevalence of HAIs and inappropriate usage of antibiotics throughout the Philippines [3, 15–17], which likely introduce external factors into an institutional work system where infection surveillance, laboratory testing, and disease management are otherwise very consistent (External Environment in Fig. 1). The overall purpose and processes of the infection control executive committee were well-received by healthcare providers, likely because the long-standing policies have become a normal part of the hospital’s culture during the past thirty years. Previous studies support this notion, showing that institutional etiquette and social norms can influence overall compliance with infection control programs [18].

The facility’s infection control nursing staff is another asset that was acknowledged to have a positive impact on patient outcomes. The six-person team at this 800-bed hospital surpasses the Centers for Disease Control recommendation of at least one full-time infection control staff for the first 100 beds, and another staff member for each additional 250 beds [19]. Infection control staff frequently utilize one of the tool-level factors identified in our study, the closed circuit television system, for hand hygiene surveillance. However, several providers felt that reporting the results of compliance data to large groups was ineffective for improving hand hygiene at the individual level. Implementing a monitor display may prove useful, as previous video surveillance interventions have found that continuously displaying the results of hand hygiene behavior on a monitor can yield a sustained improvement in compliance rates [20, 21].

Our study found that the prominent monitor display of RFID badge data in the hospital’s ICU may be an effective way to ensure individual accountability for hand hygiene compliance. One weakness of this system is the use of a shared ‘guest’ badges by all clinicians who visit the ICU from other departments. This could be addressed by providing regularly visiting providers with their own badges. While this type of system can provide powerful feedback to providers, implementing the badges throughout an entire institution could be cost-prohibitive [22]. Acquiring new equipment can be difficult in a resource-constrained facility, especially since increased patient charges are often the primary means of covering such costs. This was a concern among nurses in the discussion of medical trolley purchases. While trolleys are a useful tool in the airborne isolation unit, their absence in other floors is a barrier to hand hygiene compliance. Purchasing additional trolleys would likely be less costly than adding more RFID badges. Prioritizing the purchase of new trolleys at an organizational level, rather than on a ward-by-ward basis, could rapidly improve hand hygiene feasibility for healthcare workers.

The high turnover of nursing staff is also concerning, as it necessitates constant training and use of educational resources. A systematic review of nurses’ motivational factors in numerous developing countries identified key factors for successful retention packages [23]. In addition to financial incentives, these packages must include ways of strengthening healthcare workers’ motivation through personal recognition and career development opportunities.

Another reported barrier was the low hand hygiene compliance of attending physicians compared to nurses, a trend that is consistent with numerous institutions worldwide [24, 25]. One potential reason for this is the minimal time physicians have between patients during rounds. As several subjects suggested, providing personal portable dispensers of alcohol-based hand rub is potentially low-cost, time-saving, and would also prevent theft concerns. This type of intervention has been successful in other facilities, resulting in up to a 64% increase in hand hygiene compliance [26–28].

Implementing a verbal reminder process for hand hygiene could be another helpful practice. Several interviews suggested having the providers with the best compliance, the nurses, remind others to perform hand hygiene. This potentially nurse-driven intervention would need to account for suboptimal communication within the hierarchy of health professionals. Previous efforts to improve interprofessional collaboration have highlighted the importance of senior doctors and nurses setting an example for more junior healthcare workers, and encourage the development of shared mental models [29, 30]. This could be fostered through increasing collaborative practice, interprofessional patient rounds, or implementing a communication skills training [31]. Increasing open communication between nurses and physicians is crucial for patient safety, and interventions based on shared accountability models have had favorable impacts on hand hygiene adherence and rates of HAIs [32, 33].

Our study had several limitations. It was conducted at a single, private hospital that is considered one of the pioneers of infection control in Manila. Private hospitals comprise 60% of the roughly 1800 hospitals in the Philippines, and generally serve patients who can afford fee-for-service payments [34]. Thus, our findings may not be generalizable to smaller, community hospitals located in more rural areas of the country or to institutions that lack organizational support for infection control policies. In our institution, for example, the rate of ESBL *Klebsiella pneumoniae* based on a hospital antibiogram in 2016 for non-ICU and ICU patients was between 16 and 19% (*n* = 125), compared to a much higher rate of 40% (*n* = 8861) among 24 surveillance sites all across the Philippines. Similarly, the rate of carbapenem resistant *Acinetobacter baumanni,* though very high at 27–34% (*n* = 116), was still lower than the 52.1% (*n* = 3967) found in these surveillance hospitals [35].

The study population was limited to a small size and selected based on convenience sampling. While we sought to include participants representing a wide range of clinical experiences, our results may not reflect hospital-wide opinions regarding infection control. Other key stakeholders, such as patients, hospital management, and environmental cleaning staff may have additional perceptions and should be included in future studies.

These limitations notwithstanding, our study findings have implications for infection preventionists, hospital epidemiologists, and clinicians in resource-constrained settings. For example, the emphasis on and interest in hand hygiene compliance monitoring at our study site suggests that interventions to optimize hand hygiene might be a high priority, even in low-resource settings. Moreover, our systems approach may serve as an exemplar for other facilities seeking to prioritize infection control resources.

Previously studied infection control interventions in the Philippines have either demonstrated minimal impact, or have examined only a single disease outcome (catheter-associated urinary tract infection) [36, 37]. These studies suggest that infection control interventions in this country have the potential for success, but are also faced with the inherent difficulties of resource-limited settings. The perceived availability of resources is another challenging aspect of intervention implementation; even if resources do exist within a healthcare facility, they will not be useful if clinicians are unaware of them or do not believe they are readily available [5]. In recognition of these concerns, we incorporated the perceptions held by key stakeholders in order to prioritize areas for future intervention.

## Conclusions

Discussions with healthcare providers revealed that infection control practices in a resource-limited setting were perceived positively by most. Primary facilitators in this institution included a well-established infection control unit with support from the rest of the healthcare team and hospital organization. There are several viable opportunities for future intervention to overcome the existing barriers. These include real-time feedback of hand hygiene surveillance data, provision of medical trolleys and portable alcohol-based hand rub dispensers, improvement of retention packages for nursing staff, and advancement of interprofessional communication. These measures may provide important tools for reducing HAIs in this type of resource-limited healthcare facility.

## Abbreviations

CCTV: Closed circuit television
HAI: Hospital-acquired infection
HICEC: Hospital Infection Control Executive Committee
ICU: Intensive care unit
MDRO: Multi-drug resistant organism
RFID: Radio-frequency identification
SEIPS: Systems Engineering Initiative for Patient Safety
